# PD-1 inhibitor-augmented HAIC-TKI therapy in hepatocellular carcinoma with portal vein tumor thrombosis: real-world survival benefits, safety, and subgroup-specific efficacy

**DOI:** 10.3389/fimmu.2025.1602031

**Published:** 2025-06-12

**Authors:** Fei Cao, Chunyong Wen, Yujia Wang, Hongtong Tan, Shaohuan Hao, Jinbin Chen, Shuanggang Chen, Lujun Shen, Lin Xie, Han Qi, Tao Huang, Yaojun Zhang, Zilin Huang

**Affiliations:** ^1^ Department of Minimally Invasive and Interventional Therapy, Sun Yat-sen University Cancer Center, Guangzhou, China; ^2^ State Key Laboratory of Oncology in South China, Sun Yat-sen University, Guangzhou, China; ^3^ Guangdong Provincial Clinical Research Center for Cancer, Sun Yat-sen University, Guangzhou, China; ^4^ Collaborative Innovation Center for Cancer Medicine, Sun Yat-sen University Cancer Center, Guangzhou, China; ^5^ Department of Gastrointestinal Oncology, First People’s Hospital of Korla, Korla, China; ^6^ Department of Hepatobiliary Surgery, Sun Yat-sen University Cancer Center, Guangzhou, China

**Keywords:** PD-1 inhibitor, hepatic arterial infusion chemotherapy, tyrosine kinase inhibitors (TKIs), portal vein tumor thrombosis, hepatocellular carcinoma

## Abstract

**Background:**

PD-1/PD-L1 inhibitors have shown efficacy in improving the prognosis of patients with hepatocellular carcinoma (HCC) accompanied by portal vein tumor thrombosis (PVTT) in pivotal clinical trials including the landmark IMbrave150 study. However, not all the patients benefit from the PD-1/PD-L1 blockade immunotherapy. This study aimed to improve the identification of PVTT-associated HCC patients who may benefit from the combination of PD-1 inhibitor and hepatic arterial infusion chemotherapy (HAIC) and tyrosine kinase inhibitor (TKI) treatment under real-world conditions.

**Methods:**

From 377 HCC-PVTT patients receiving HAIC-TKI ± PD-1 inhibitors (2016-2023), we compared 76 dual-therapy (HT) and 175 triple-therapy (HTP) cases. Median follow-up period was 34.8 months in the HT group and 33.4 months in the HTP group (*P*=0.175). Propensity score matching (1:1 caliper=0.2) was used to balance baseline characteristics. Overall survival (OS), progression-free survival (PFS), objective response rate (ORR), and safety were evaluated in both groups. Specific subgroups including Vp4 type PVTT, extrahepatic metastases, and patients over 60 years old, were analyzed.

**Results:**

Triple therapy significantly improved median OS (24.6 vs. 13.5 months; HR=0.58, 95%CI:0.42–0.80; *P*=0.001) and PFS (11.1 vs. 6.4 months; HR=0.56, *P*<0.001), with a 15% absolute ORR increase (66.3% vs. 51.3%, *P*=0.034). In subgroup analysis, for patients with Vp4 type PVTT, the addition of PD-1 inhibitor prolonged overall survival by 6.0 months (*P*=0.04). For patients aged 60 years and above, the addition of PD-1 inhibitor prolonged overall survival by 1.9 months (*P*=0.363). For patients with extrahepatic metastasis, the addition of PD-1 inhibitor prolonged overall survival by 3.0 months (*P*=0.913). Grade 3–4 adverse events were comparable (30.9% vs. 19.7%, *P*=0.09), but two patients experienced immune treatment-related fatalities in the HTP group.

**Conclusion:**

The triple therapy (HAIC-TKI-PD-1) demonstrated superior efficacy over HAIC-TKI dual therapy in HCC patients with PVTT, achieving significant improvements in ORR, mOS, and mPFS, with an acceptable safety profile. However, PD-1 inhibitors showed minimal survival benefits in patients aged >60 or with extrahepatic metastases.

## 1 Introduction

Hepatocellular carcinoma (HCC), ranking as the sixth most prevalent malignancy globally and the third primary contributor to cancer-related mortality, accounts for over 750,000 annual deaths worldwide (1). A significant portion of HCC patients are diagnosed with portal vein tumor thrombosis (PVTT), making curative treatments impractical (2, 3). The median survival time for untreated PVTT patients is 2.7-4.0 months (4). Advanced HCC treatments have evolved significantly, moving from surgery, radiation, and chemotherapy to more targeted and individualized approaches like immunotherapy (5). But immunotherapy is not effective for all HCC patients. Selecting the appropriate population ensures more precise treatment and fewer complications due to treatment (6).

In the 2022 BCLC staging and treatment strategy, atezolizumab-bevacizumab and durvalumab-tremelimumab are recommended as first-line treatment options for patients with advanced hepatocellular carcinoma (7–9). However, the combination of atezolizumab plus bevacizumab only showed limited benefit in HCC patients with Vp4 stage PVTT, with a median overall survival of only 7.6 months (10, 11). The HIMALAYA study evaluated the efficacy of tremelimumab plus durvalumab. Significant improvement in overall survival was shown in non-resectable hepatocellular carcinoma patients. However, the study excluded HCC patients with portal vein thrombosis, as these patients typically have a poorer prognosis (12). Hepatic arterial infusion chemotherapy (HAIC) with oxaliplatin, fluorouracil, and leucovorin has shown efficacy in the treatment of advanced HCC, with lower toxicity (13). The FOHAIC-1 trial demonstrated that HAIC-FOLFOX regimen significantly improved survival outcomes in advanced HCC patients compared to sorafenib (13–15). Furthermore, a phase III trial showed that the combination of sorafenib and HAIC-FOLFOX improved ORR, PFS, and OS compared to sorafenib monotherapy in advanced HCC (15). The combination of HAIC and TKI has shown superior efficacy over TKI alone in the treatment of HCC with portal vein tumor thrombosis (16).

For patients with PVTT, current research is investigating the inclusion of PD-1 inhibitors in HAIC-FOLFOX and TKI treatment regimens to further improve survival outcomes (17). However, the results of these phase III clinical trials have not yet been published. Additionally, the efficacy of the combination of TKI and PD-1 inhibitors for patients with advanced hepatocellular carcinoma is inconsistent (18, 19). Although phase II clinical trials showed promising results for the combination of pembrolizumab and lenvatinib, the subsequent phase III clinical trial Leap 002 did not demonstrate a significant improvement in survival (19). In China, the triple therapy of HAIC-FOLFOX, TKI, and PD-1 inhibitors is commonly used to treat advanced hepatocellular carcinoma. Our department’s phase II clinical trial found that the combination of HAIC-FOLFOX, rivoveranib (a TKI), and camrelizumab (a PD-1 inhibitor) achieved an 88.6% objective response rate in patients with advanced hepatocellular carcinoma (20). Further phase III clinical trials are still needed to determine whether PD-1 inhibitors can improve the efficacy of HAIC and TKI in PVTT hepatocellular carcinoma patients. This triple therapy has been widely applied to PVTT hepatocellular carcinoma patients (20), but it is unclear whether PD-1 inhibitors are necessary for specific subgroups, such as patients with Vp4, poor liver function, age over 60, or extrahepatic metastases.

This study aims to compare the safety and efficacy of HAIC and TKI in combination with PD-1 inhibitors versus HAIC and TKI alone in HCC patients with PVTT, and to confirm whether PD-1 inhibitors can bring survival benefits to specific subgroups.

## 2 Materials and methods

### 2.1 Study design and patients

The study protocol was approved by the Institutional Review Board of Sun Yat-sen University Cancer Center (B2025-329–01). which waived the need for written informed consent due to the retrospective nature of the study. The study was performed in accordance with the Declaration of Helsinki. HCC was diagnosed following the stringent protocols outlined by the American Association for the Study of Liver Disease (21).

The imaging features of PVTT exhibit distinct solid masses within the portal vein throughout the various phases of intravenous contrast-enhanced three-phase computed tomography. Imaging features of PVTT are similar to those of HCC. It is important to differentiate PVTT from portal vein thrombosis, as the latter does not show enhancement in the arterial phase (3).

Consecutive patients were identified via the electronic medical records based on the following criteria: (a) histologically or clinically confirmed diagnosis of HCC with PVTT; Clinically diagnosis refers to a diagnosis established through non-invasive imaging criteria combined with characteristic clinical and laboratory findings (21); (b) first-line treated with HAIC- FOLFOX6 and TKIs including sorafenib, lenvatinib or apatinib; (c) at least one measurable intrahepatic lesion according to the modified Response Evaluation Criteria in Solid Tumors (mRECIST) (22). The exclusion criteria included the following: (a) incomplete medical information; (b) prior treatment for HCC; (c) inadequate organ function of kidney, heart, and lung; (d) history of secondary malignancy.

### 2.2 Treatment protocol

HAIC was suggested to be performed every 3 weeks. All patients received an artery catheter procedure guided by digital subtraction angiography. Angiography of the celiac trunk and superior mesenteric artery were performed to find out the blood supply to the tumor. Combined with preoperative CT, MRI or DSA images, the microcatheter was inserted into the corresponding target vessel. Commonly selected sites for microcatheter placement include the left hepatic artery, right hepatic artery, and proper hepatic artery. If the gastroduodenal artery cannot be avoided, the coil was used to embolize the gastroduodenal artery to prevent drug shunting or gastrointestinal side effects. In instances a portion of the blood supply to the tumor was not derived from celiac trunk, additional investigation via phrenic artery and intercostal artery angiography might be required. Tumors supplied by the phrenic artery or intercostal artery were treated with embolization or trans-arterial chemotherapy. Following placement of the catheter and microcatheter, the prescribed regimen was administered: oxaliplatin 85 mg/m^2^ (hour 0 to 3 on day 1); leucovorin 400 mg/m^2^ (hour 3 to 4 on day 1); 5-fluorouracil 400 mg/m^2^ bolus (hour 4 to 5 on day 1); and 5-FU 2400 mg/m^2^ over 46 h on day 1 and 2 (23). In situations where a patient exhibited moderate to severe ascites, a decline in white blood cell or platelet counts exceeding grade 3 that remained unresponsive to therapeutic intervention, severe vomiting, or abdominal pain surpassing grade 3, it was recommended to halt the administration of HAIC. Notably, elevated concentrations of liver enzymes and bilirubin did not constitute contraindications for HAIC.

TKIs were started 3 days after the first HAIC and continued thereafter. Sorafenib was administered at a dosage of 400 mg twice daily, lenvatinib at a dosage of 8 mg daily for individuals weighing 60 kg or less, or 12 mg daily for those weighing over 60 kg, or apatinib at a dosage of 250 mg daily (11). Patients discontinued the use of TKI drugs during arterial infusion chemotherapy to mitigate the exacerbation of symptoms induced by oxaliplatin and 5-FU. Patients were recommended to resume TKI drug intake for the duration of the treatment period. PD-1 antibodies, such as sintilimab (200 mg every three weeks), tislelizumab (200 mg every three weeks), or toripalimab (240 mg every three weeks), were administered on the first day following arterial infusion chemotherapy. Following the cessation of HAIC, it was advised that both TKIs and PD-1 inhibitors continue. PD-1 inhibitors were administered once every 3 weeks. When severe TRAEs happened, corticosteroids were used.

### 2.3 Follow-up

Clinical and radiological data were retrospectively gathered from the electronic medical record. These data included variables such as age, gender, ECOG performance status, etiology, lymphocyte count, neutrophil count, platelet count, prothrombin time (PT), cholinesterase (CHE), alanine aminotransferase (ALT), aspartate aminotransferase (AST), total bilirubin (TBIL), albumin (ALB), C-reactive protein (CRP), creatinine, alpha-fetoprotein (AFP), protein induced by vitamin K absence or antagonist II (PIVKA II), tumor number, largest tumor size, tumor location, presence of metastasis, ascites, and PVTT classification (Japanese Vp classification) (24). The imaging data every 6 weeks were assessed by two radiologists according to the modified Response Evaluation Criteria in Solid Tumors (mRECIST) criteria. Tumor response was classified as complete response (CR), partial response (PR), stable disease (SD), and progressive disease (PD). The ORR was defined as the proportion of patients who achieved CR and PR. The DCR was defined as the proportion of patients who achieved CR, PR and SD. PFS was defined as the duration from the initiation of HAIC to either disease progression or death from any cause. OS was determined as the time from HAIC initiation to death from any cause. ORR was calculated as the proportion of patients showing complete or partial responses based on the mRECIST criteria. Adverse events were evaluated and graded by the National Cancer Institute Common Terminology Criteria for Adverse Events, version 5.0.

The treatment protocol and follow up were applied by a previous study group of our department in the past, and written informed consent for participation was obtained by the previous study group (20).

### 2.4 Statistical analysis

Pearson χ2 test and Fisher’s exact test was used to compare categorical variables between groups, respectively. Survival analysis was conducted by plotting Kaplan-Meier survival curves and comparing them with the log-rank test. Hazard ratios (HRs) and 95% confidence intervals (CIs) were calculated using the Cox proportional hazard model for survival analysis. The Cox’s proportional hazards regression model was employed to rigorously assess the potential influencing factors pertaining to both PFS and OS. The variables with *P* < 0.05 in the univariate analysis were included in the multivariate analysis. All *P* values were calculated using a two-sided approach, with significance defined as *P* < 0.05. Statistical analysis was conducted utilizing either SPSS version 22.0 (25) or R version 4.2.1 (The R Foundation for Statistical Computing, 2022). Propensity score matching (PSM) analysis was employed to mitigate selection bias and confounding variables, resulting in the formation of a propensity score-matched cohort (26). A multivariate logistic regression model was employed to calculate propensity scores for individual patients, followed by 1:1 matching between groups using nearest-neighbor methodology with non-replacement matching. The parameters included in the PSM process were age, sex, etiology, PVTT stage, tumor number, largest tumor size, tumor location, ECOG PS, AFP, PIVKA II, Child-Pugh stage, and metastasis. A caliper width of 0.2 standard deviations was set to prevent poor matching.

## 3 Results

### 3.1 Patient characteristics

**Figure 1:**
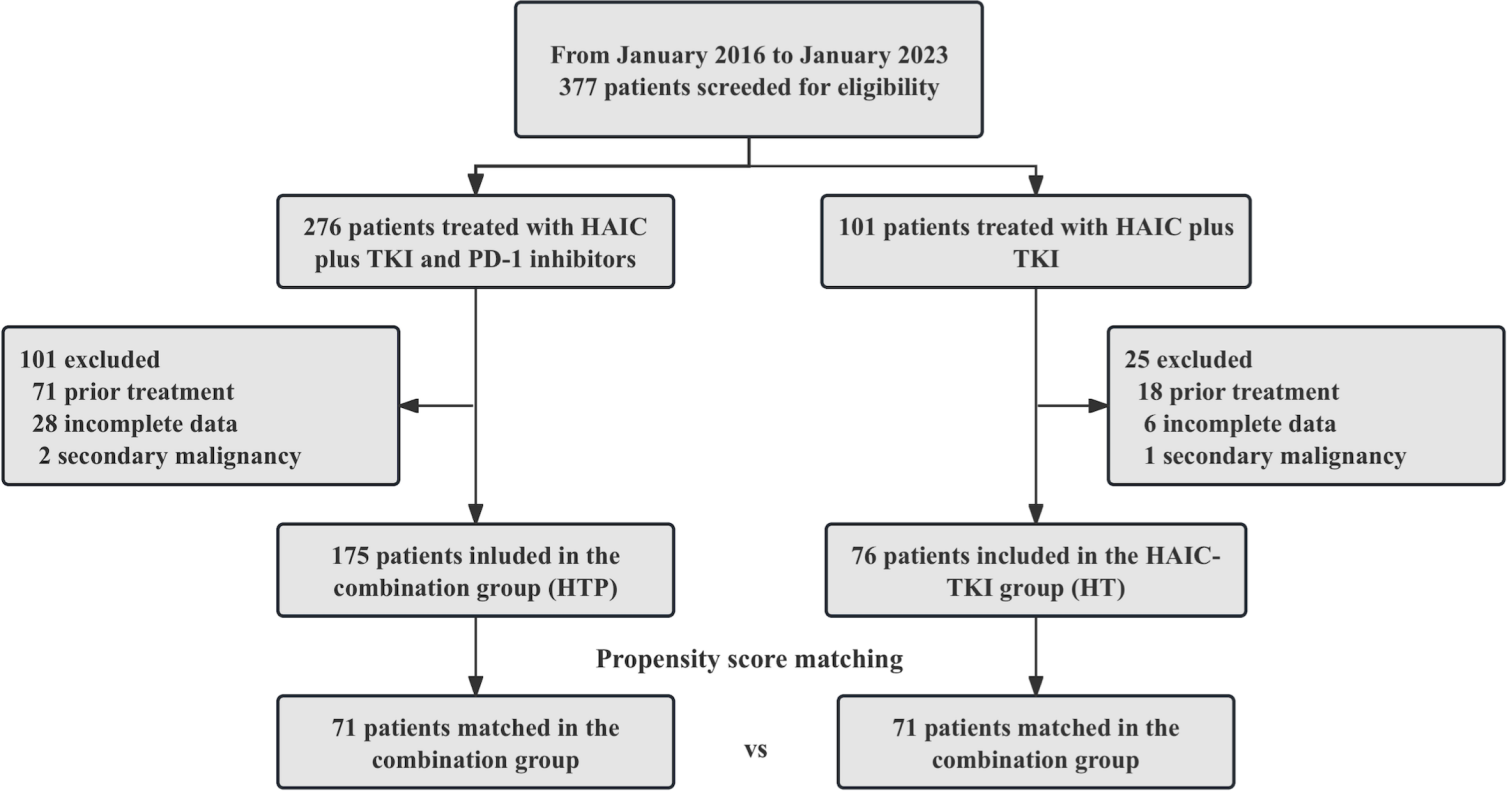
Patient selection flow.

During the period from January 2016 to January 2023, a total of 377 patients with HCC combined with PVTT who received HAIC-TKI therapy were continuously enrolled, and finally, 76 patients received dual therapy (HAIC-TKI, HT group) and 175 patients received triple therapy (HAIC-TKI-PD-1 inhibitor, HTP group) meeting the inclusion criteria (see flowchart in **Figure 1**). The mean age of the cohort was 49.5 ± 11.5 years, and 91.2% (229/251) were male. Tumor burden characteristics showed a median maximum tumor diameter of 10.8 cm, 38.2% (95/251) had extrahepatic metastases, and 38.6% (97/251) had Vp4-grade PVTT. Hepatitis B virus (HBV) infection was the main cause (89.6%), and all patients received entecavir or tenofovir antiviral therapy.

Overall, 71 patients in the HT group and 71 in the HTP group were enrolled in the PSM analysis (1:1), whereas 5 patients in the HT group and 104 in the HTP group were excluded by PSM. Characteristics including age, sex, etiology, PVTT stage, tumor number, largest tumor size, tumor location, ECOG PS, AFP, PIVKA II, Child-Pugh stage, and metastasis were matched and shown in **Table 1**. There were no significant differences in the baseline characteristics between the two groups (*P* > 0.05).

**Table 1:** Patient baseline demographic and clinical characteristics.

Characteristics	Before PSM			After PSM		
	TKI and HAIC, N=76	TKI, HAIC and PD-1 inhibitor, N=175	p-value	TKI and HAIC, N=71	TKI, HAIC and PD-1 inhibitor, N=71	p-value
Age (years)			1.0			0.614
≤ 50	40 (52.6%)	92 (52.6%)		36 (50.70%)	40 (56.34%)	
> 50	36 (47.4%)	83 (47.4%)		35 (49.30%)	31 (43.66%)	
Sex			0.627			0.778
Men	68 (89.47%)	161 (92.00%)		63 (88.73%)	65 (91.55%)	
Women	8 (10.53%)	14 (8.00%)		8 (11.27%)	6 (8.45%)	
ECOG PS			0.207*			0.605
0	69 (90.79%)	144 (82.29%)		64 (90.14%)	61 (85.92%)	
1	7 (9.21%)	30 (17.14%)		7 (9.86%)	10 (14.08%)	
2	0 (0.00%)	1 (0.57%)		0	0	
Etiology			0.557*			0.681*
HBV	72 (94.74%)	159 (90.86%)		67 (94.37%)	68 (95.77%)	
HCV	0 (0.00%)	1 (0.57%)		0 (0.00%)	1 (1.41%)	
Other	4 (5.26%)	15 (8.57%)		4 (5.63%)	2 (2.82%)	
Albumin			0.362			0.378
≥ 35 g/L	60 (78.95%)	148 (84.57%)		56 (78.87%)	61 (85.92%)	
< 35 g/L	16 (21.05%)	27 (15.43%)		15 (21.13%)	10 (14.08%)	
AFP			0.008			0.544*
≤ 20 ng/mL	7 (9.21%)	36 (20.57%)		7 (9.86%)	4 (5.63%)	
20–400 ng/mL	7 (9.21%)	31 (17.71%)		7 (9.86%)	10 (14.08%)	
> 400 ng/mL	62 (81.58%)	108 (61.71%)		57 (80.28%)	57 (80.28%)	
DCP			0.390			0.999
≤ 400 mAU/mL	18 (23.68%)	32 (18.29%)		14 (19.72%)	15 (21.13%)	
>400 mAU/mL	58 (76.32%)	143 (81.71%)		57 (80.28%)	56 (78.87%)	
Maximum tumor size			0.829			0.735*
≤ 5 cm	5 (6.58%)	14 (8.00%)		5 (7.04%)	4 (5.63%)	
5–10 cm	29 (38.16%)	60 (34.29%)		27 (38.03%)	23 (32.39%)	
> 10 cm	42 (55.26%)	101 (57.71%)		39 (54.93%)	44 (61.97%)	
Ascites			0.752*			0.844
No	59 (77.63%)	132 (75.43%)		55 (77.46%)	53 (74.65%)	
Mild to moderate	16 (21.05%)	42 (24.00%)		16 (22.54%)	18 (25.35%)	
Severe	1 (1.32%)	1 (0.57%)		0	0	
Child-Pugh classification			0.630*			0.792*
A	66 (86.84%)	156 (89.14%)		62 (87.32%)	63 (88.73%)	
B	9 (11.84%)	17 (9.71%)		9 (12.68%)	7 (9.86%)	
C	1 (1.32%)	2 (1.14%)		0 (0.00%)	1 (1.41%)	
Portal vein invasion			0.985			0.650
Vp1-2	23 (30.26%)	51 (29.14%)		23 (32.39%)	18 (25.35%)	
Vp3	23 (30.26%)	57 (32.57%)		22 (30.99%)	24 (33.80%)	
Vp4	30 (39.47%)	67 (38.29%)		26 (36.62%)	29 (40.85%)	
No. of tumors			1			0.854
Single	23 (30.26%)	53 (30.29%)		22 (30.99%)	20 (28.17%)	
Multiple	53 (69.74%)	122 (69.71%)		49 (69.01%)	51 (71.83%)	
Tumor location			0.836			0.954
Left lobe	7 (9.21%)	17 (9.71%)		6 (8.45%)	7 (9.86%)	
Right lobe	38 (50.00%)	83 (47.43%)		34 (47.89%)	34 (47.89%)	
Both lobes	31 (40.79%)	75 (42.86%)		31 (43.66%)	30 (42.25%)	
Extrahepatic metastasis			0.888			0.604
Yes	28 (36.84%)	67 (38.29%)		25 (35.21%)	29 (40.84%)	
No	48 (63.16%)	108 (61.71%)		46 (64.79%)	42 (59.16%)	

*Fisher’s Exact Test.

### 3.2 Treatment mode differences

In the HT group, 39.5% (30/76) received 1–2 HAIC (median 3 [IQR: 2-6] times, **Table 1**), 56.8% (43/76) received 3–6 times, and 4.0% (3/76) received 7–8 times. In the HTP group, 20.6% (36/175) received 1–2 HAIC (median 5 times), 70.3% (123/175) received 3–6 times, and 9.1% (16/175) received 7–8 times. The distribution of first-line TKI drugs was: lenvatinib 64.1% (161/251), sorafenib 14.3% (36/251), and apatinib 21.5% (54/251).

### 3.3 Tumor response

Based on mRECIST criteria (22), 155 patients (61.75%) achieved an objective response, with 40 patients (15.9%) achieving a complete response. Patients in the HTP group exhibited a higher ORR compared to those in the HT group (66.3% vs. 51.3%, *P*=0.034). CR was observed in 34 patients and six patients (19.4% vs. 7.9%) in the HTP group and the HT group. PR was achieved in 82 patients (46.9%) in the HTP group and 33 patients (43.4%) in the HT group. After PSM, patients in the HTP group maintained a higher ORR than those in the HT group (69.0% vs. 50.7%, *P*=0.04). CR was achieved in 15 patients (21.1%) in the HTP group and five patients (7.0%) in the HT group. PR was achieved in 34 patients (47.9%) in the HTP group and 31 patients (43.7%) in the HT group (**Table 2**).

**Table 2:** Summary of best response.

Tumor Response	Before PSM				After PSM			
	Overall, N=251	TKI and HAIC, N=76	TKI, HAIC and PD-1 inhibitor, N=175	*p*-value	Overall, N=142	TKI and HAIC, N=71	TKI, HAIC and PD-1 inhibitor, N=71	*p*-value
Best response				0.008				0.007
Complete response	40 (15.94%)	6 (7.9%)	34 (19.43%)		20 (14.08%)	5 (7.04%)	15 (21.13%)	
Partial response	115 (45.82%)	33 (43.42%)	82 (46.86%)		65 (45.77%)	31 (43.66%)	34 (47.89%)	
Stable disease	56 (22.31%)	22 (28.95%)	34 (19.43%)		33 (23.24%)	20 (28.17%)	13 (18.31%)	
Progressive disease	38 (15.14%)	13 (17.11%)	25 (14.29%)		22 (15.49%)	13 (18.31%)	9 (12.68%)	
Not evaluable*	2 (0.80%)	2 (2.63%)	0		2 (1.41%)	2 (2.82%)	0	
Objective response rate	155 (61.75%)	39 (51.32%)	116 (66.29%)	0.034	85 (59.86%)	36 (50.70%)	49 (69.01%)	0.04

Unless otherwise indicated, data are numbers of patients, and data in parentheses are percentages. HAIC, hepatic arterial infusion chemotherapy; mRECIST, modified Response Evaluation Criteria in Solid Tumors; *No postbaseline assessment available for response evaluation.

### 3.4 Survival results

Median follow-up period was 34.8 months (range, 1.0-57.0 months) in the HT group and 33.4 months (range, 0.7-94.4 months) in the HTP group (*P*=0.175). During the follow-up period, 60 patients (78.9%) in the HT group and 100 patients (57.1%) in the HTP group died (*P*=0.001). Tumor progression was observed 68 patients (89.5%) in the HT group and 126 patients (72.0%) in the HTP group.

#### 3.4.1 Survival results before PSM

The median OS was 13.5 months (95% CI: 10.7-16.3 months) for the HT group and 24.6 months (95%CI: 18.0-31.2 months) for the HTP group, with a hazard ratio (HR) of 0.58 (95% CI, 0.42-0.80; *P*=0.001) for death. The 1-, 2-, and 3-year overall survival rates in the HT and HTP groups were 54.7% vs 71.4%, 23.0% vs 48.7%, and 19.0% vs 35.3%, respectively. The median PFS was 6.4 months (95% CI: 4.7-8.1 months) for the HT group and 11.1 months (95% CI: 7.7-14.4 months) for the HTP group, yielding a hazard ratio of 0.56 (95% CI, 0.42 to 0.76; *P* < 0.001) for disease progression or death.

#### 3.4.2 Survival results after PSM

**Figure 2:**
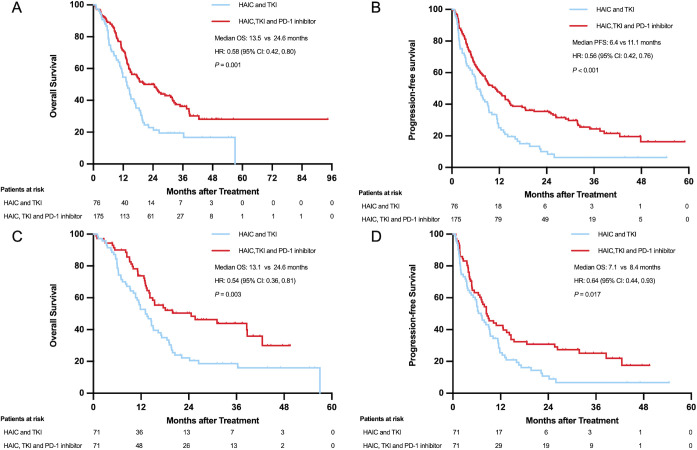
Overall survival and progression-free survival before **(A, B)** and after PSM **(C, D)**.

The median OS in the HT and HTP groups were 13.1 months (95% CI: 10.0-16.2 months) and 24.6 months (95% CI: 7.8-41.4 months) (hazard ratio for death, 0.54; 95% CI, 0.36 to 0.81; *P* = 0.003), respectively. The 1-, 2-, and 3-year overall survival rates in the HT and HTP groups were 52.9% vs 73.7%, 22.2% vs 48.9%, and 18.2% vs 41.9%, respectively. The median PFS for patients was 6.2 months (95% CI: 2.4-10.0 months) for the HT group and 7.9 months (95% CI: 4.4-11.4 months) for the HTP group, yielding a hazard ratio of 0.68 (95% CI, 0.38 to 1.23; *P=* 0.2) for disease progression or death (**Figure 2**).

### 3.5 Subgroup analysis

**Figure 3:**
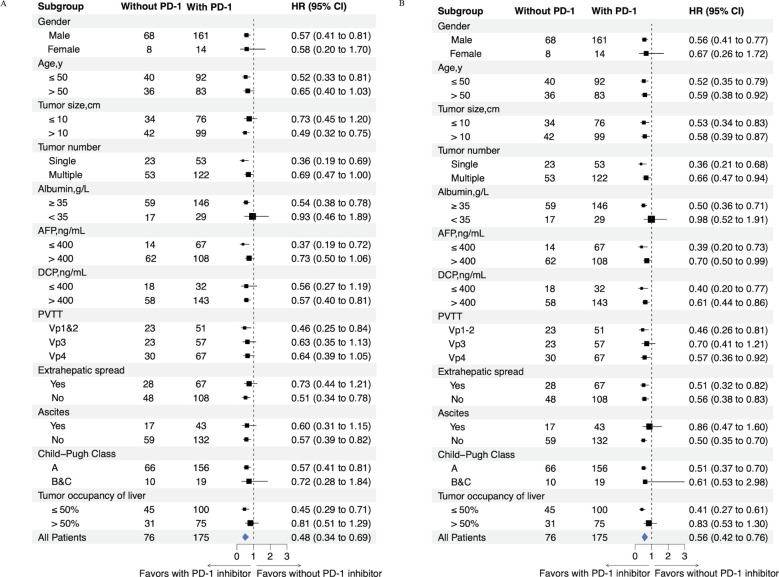
Forest plot shows factors associated with OS **(A)** and PFS **(B)** in patients who received HAIC-TKI-PD-1 or HAIC-TKI.

Survival benefits of PD-1 inhibitors demonstrated significant heterogeneity, as shown in **Figure 3**. PSM and subgroup analyses were implemented to address confounding factors.

**Figure 4:**
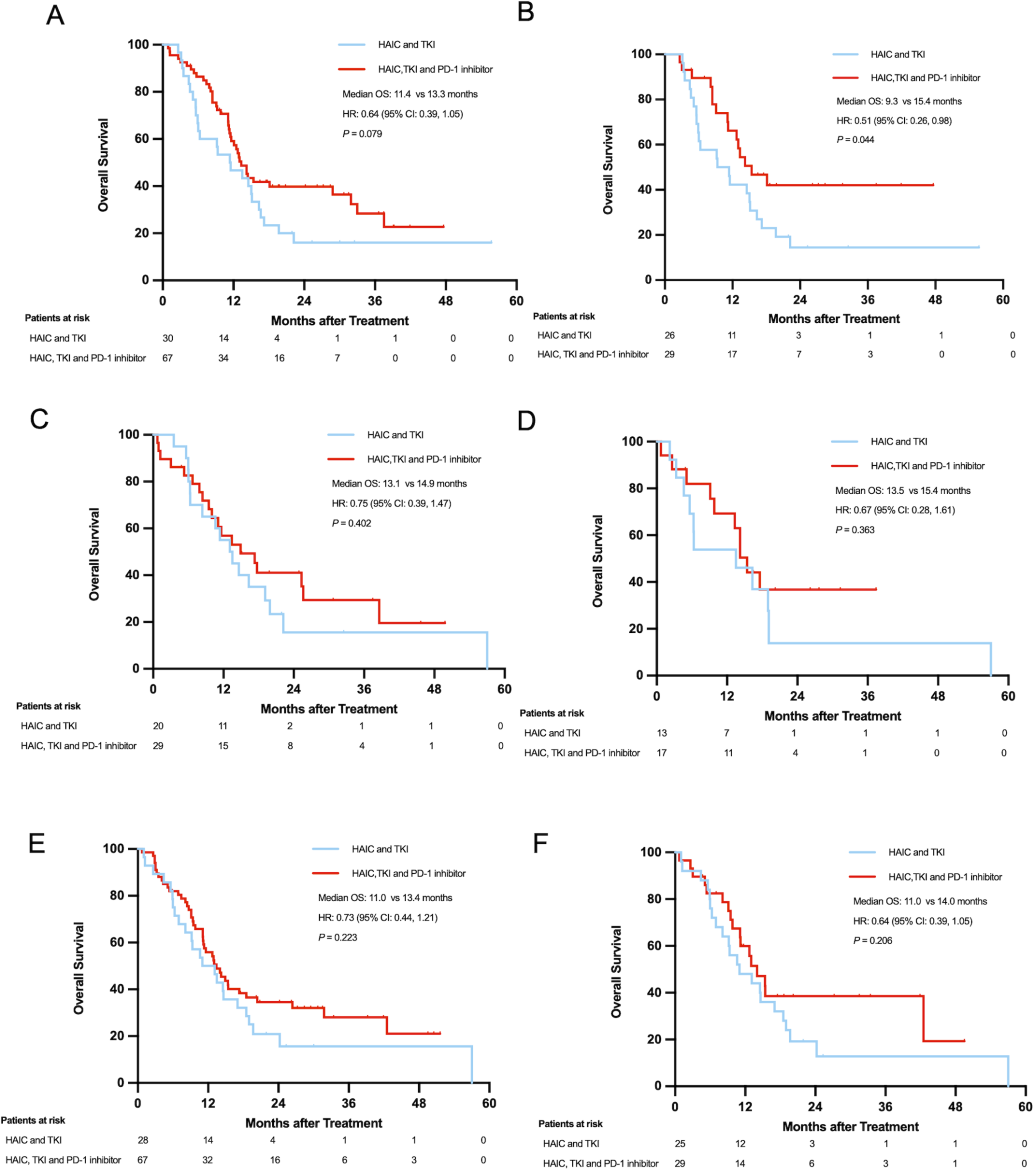
Overall survival of patients with PVTT Vp4 before **(A)** and after PSM **(B)**; Overall survival of patients over 60 years old before **(C)** and after PSM **(D)**; Overall survival of patients with extrahepatic metastases before **(E)** and after PSM **(F)**.

In Vp4 PVTT patients, pre-PSM analysis showed a trend toward improved survival in the HTP group (13.3 vs. 11.4 months; HR=0.64; 95% CI: 0.39-1.05; *P*=0.076), which became statistically significant post-PSM (15.3 vs. 9.3 months; HR=0.51; 95% CI: 0.26-0.98; *P*=0.040). Conversely, Vp3 PVTT subgroups exhibited no significant survival differences either pre-PSM (24.6 vs. 18.5 months; HR=0.63; 95% CI: 0.35-1.13; *P*=0.119) or post-PSM (24.6 vs. 13.1 months; HR=0.62; 95% CI: 0.31-1.26; *P*=0.184). The most pronounced HTP advantage emerged in Vp1–2 PVTT patients, with significant survival benefits both pre-PSM (27.2 vs. 14.6 months; HR=0.46; 95% CI: 0.25-0.84; *P*=0.010) and post-PSM (31.1 vs. 14.6 months; HR=0.43; 95% CI: 0.19-0.98; *P*=0.039) (**Figures 4A, B**).

Age stratification revealed distinct patterns: patients ≤60 years showed consistently superior survival with HTP therapy (pre-PSM: 24.6 vs. 13.8 months; HR=0.56; 95% CI: 0.39-0.82; *P*=0.002; post-PSM: 25.6 vs. 13.1 months; HR=0.50; 95% CI: 0.31-0.80; P=0.003), while older patients (>60 years) demonstrated no significant differences (pre-PSM: 14.9 vs. 13.1 months; HR=0.75; 95% CI: 0.39-1.47; *P*=0.402; post-PSM: 15.4 vs. 13.5 months; HR=0.67; 95% CI: 0.28-1.61; *P*=0.363) (**Figures 4C, D**).

Metastatic status significantly influenced outcomes. Patients with extrahepatic metastases showed comparable survival between groups (pre-PSM: 13.4 vs. 11.0 months; HR=0.73; 95% CI: 0.44-1.21; *P*=0.220; post-PSM: 14.0 vs. 11.0 months; HR=0.66; 95% CI: 0.35-1.26; *P*=0.206). In contrast, non-metastatic patients exhibited marked HTP survival advantages (pre-PSM: 28.8 vs. 14.6 months; HR=0.51; 95% CI: 0.34-0.78; *P*=0.002; post-PSM: 31.1 vs. 13.8 months; HR=0.47; 95% CI: 0.27-0.802; *P*=0.005) (**Figures 4E, F**).

### 3.6 Prognostic factors associated with OS and PFS

**Table 3** displays the results of univariate and multivariate analyses conducted on predictors for OS and PFS in the entire cohort.

**Table 3:** Univariate and multivariate analysis of overall survival and progression-free survival.

Univariate and multivariate analysis for predictors of OS and PFS								
Variables	Overall survival				Progression-free survival			
	Univariate analysis HR (95% CI)	*P*	Multivariate analysis HR (95% CI)	*P*	Univariate analysis HR (95% CI)	*P*	Multivariate analysis HR (95% CI)	*P*
Age,y (> 50 vs ≤50)	0.938 (0.687-1.281)	0.687			0.829 (0.625-1.101)	0.195		
Sex (Male vs Female)	0.946 (0.556-1.611)	0.838			0.792 (0.493-1.273)	0.335		
Etiology (HBV vs others)	0.987 (0.534-1.822)	0.966			1.063 (0.616-1.833)	0.827		
PLT, 10^9^/L (≤100 vs >100)	3.065 (1.135-8.276)	0.027	3.167 (1.167-8.595)	0.024	0.496 (0.233-1.056)	0.069		
PT, sec (>13.5 vs ≤ 13.5)	1.652 (1.129-2.417)	0.01			1.494 (1.056-2.112)	0.023		
CHE, U/L (≤5000 vs > 5000)	1.443 (1.051-1.982)	0.023			1.429 (1.072-1.905)	0.015		
ALT, U/L (>50 vs ≤50)	1.477 (1.082-2.016)	0.014			1.426 (1.075-1.892)	0.014		
AST, U/L (>40 vs ≤40)	1.987 (1.229-3.214)	0.005	1.818 (1.110-2.977)	0.018	1.470 (0.978-2.208)	0.064		
TBIL, μmol/L, (>51.3 vs ≤51.3)	1.354 (0.599-3.064)	0.467			1.389 (0.684-2.822)	0.363		
ALB, g/L (≤30vs > 30)	3.932 (1.905-8.118)	0.001	3.896 (1.870-8.120)	0.001	2.651 (1.350-5.206)	0.005	2.498 (1.248-5.000)	0.010
CRP, mg/L (>3 vs ≤3)	1.384 (0.847-2.262)	0.195			1.413 (0.8**97**-2.223)	0.136		
AFP, ng/mL (>400 vs ≤400)	1.507 (1.067-2.129)	0.020			1.645 (1.204-2.246)	0.002	1.611 (1.164-2.230)	0.004
PIVKA II, ng/ml (>400 vs ≤400)	0.736 (0.497-1.091)	0.127			0.889 (0.625-1.263)	0.510		
Tumor number (multiple vs single)	1.752 (1.219-2.519)	0.002	1.557 (1.076-2.253)	0.019	1.756 (1.268-2.431)	0.001	1.857 (1.337-2.578)	<0.001
Tumor size, cm (>10 vs ≤10)	1.298 (0.949-1.776)	0.103			0.705 (0.529-0.939)	0.062		
Metastasis (Yes vs no)	1.609 (1.173-2.207)	0.003	1.564 (1.135-2.155)	0.006	1.451 (1.087-1.936)	0.012		
Ascites (Yes vs No)	1.629 (1.140-2.327)	0.007			1.535 (1.109-2.125)	0.01		
PVTT Vp (3&4 vs1&2)	1.384 (0.977-1.960)	0.067			1.085 (0.793-1.483)	0.611		
Child-Pugh (B&C vs A)	1.611 (0.996-2.605)	0.052			1.456 (0.949-2.236)	0.086		
NLR (>2.5 vs ≤ 2.5)	1.442 (1.011-2.058)	0.043			1.486 (1.074-2.056)	0.017	1.487 (1.069-2.067)	0.018
Treatment (HTP vs HP)	0.580 (0.421-0.801)	0.001	0.627 (0.454-0.866)	0.005	0.564 (0.418-0.761)	0.000	0.630(0.460-0.861)	0.004

NLR, neutrophil-to-lymphocyte ratio.

The multivariate analysis identified several independent predictive factors for OS, including PLT, AST, ALB, tumor number, metastasis, and PD-1 inhibitor treatment (HR=0.63; 95% CI: 0.45-0.87, *P* =0.005). The results of the multivariate analysis indicated that several independent predictive factors were significantly associated with PFS, including ALB, AFP, tumor number, NLR, and PD-1 inhibitor treatment (HR=0.63; 95% CI: 0.46-0.86, *P* =0.004).

### 3.7 Subsequent treatment

14 patients underwent surgical resection, 31 patients underwent local thermal ablation, 6 patients underwent SBRT, and the remaining 200 patients continued with drug maintenance treatment. Overall survival was significantly longer with surgical resection (HR for death, 0.276; 95%CI: 0.113-0.674), local thermal ablation (HR for death, 0.163; 95%CI: 0.076-0.349), and SBRT (HR for death, 0.483; 95%CI: 0.178-1.310) compared with drug maintenance. Among the cohort of 155 patients who attained clinical remission, 108 patients opted for ongoing medical therapy, 14 underwent surgical intervention, 28 received local ablation therapy, and 5 patients underwent SBRT. Among the 115 patients who experienced a partial response, eight patients (7.0%) underwent surgical resection, seven patients (6.1%) received local ablation therapy, three patients (2.6%) received SBRT, and the majority of patients (n=97, 84.4%) continued with medical treatment. In contrast, out of the 40 patients who achieved complete response, six patients (15.0%) underwent surgical resection, 21 patients (52.5%) received local ablation therapy, two patients (50.0%) received stereotactic body radiation therapy (SBRT), and 11 patients (27.5%) continued with medical treatment.

### 3.8 Safety

The overall incidences rates of adverse events of any grade in the HTP and HT groups were 158 (90.3%) and 66 (86.8%), respectively (**Table 4**). The numbers of Grade 3 or 4 adverse events occurred in the HTP and HT groups were 54 (30.9%) and 15 (19.7%), respectively. There was no statistically significant difference in the incidence of any grade (*P*=0.51) and grade 3–4 adverse events (*P*=0.09) between the two group. Although the difference in values did not reach statistical significance, there is a need for clinical vigilance for higher risk of toxicity.

**Table 4:** Treatment-related adverse events.

Adverse Events				
Categories	TKI and HAIC, N=76		TKI, HAIC and PD-1 inhibitor, N=175	
Event (%)	Any Grade	Grade 3-4	Any Grade	Grade 3-4
Any adverse event	66(86.84%)*	15(19.73%)#	158(90.29%)*	54(30.85%)#
Leukopenia	6(7.89%)	1(1.32%)	27(15.43%)	3(1.71%)
Neutropenia	9(11.84)	1(1.32%)	45(25.71%)	17(9.71%)
Thrombocytopenia	22(28.95)	1(1.32%)	52(29.71%)	5(2.86%)
Anemia	0	0	5(2.86%)	0
Elevated AST/ALT	32(42.11%)	6(7.89%)	78(44.57%)	17(9.71%)
Hypoalbuminemia	16(21.05%)	0	37(21.14%)	0
Elevated bilirubin	6(7.89%)	0	20(11.43%)	1(0.57%)
Abdominal pain	7(9.21%)	1(1.31%)	16(9.14%)	4(2.29%)
Hypothyroidism	0	0	6(3.43%)	0
Nausea	8(10.53%)	2(2.63%)	16(9.14%)	3(1.71%)
Gastroduodenal ulcer	1(1.31%)	1(1.31%)	3(1.71%)	3(1.71%)
Diarrhea	3(3.94%)	1(1.31%)	5(2.86%)	0
Fatigue	5(6.59%)	1(1.31%)	5(2.86%)	2(1.14%)
Peripheral neuropathy	1(1.31%)	0	1(0.57%)	1(0.57%)
Fever	8(10.53%)	2(2.63%)	22(12.57%)	2(1.14%)
Immune-mediated liver injury	0	0	2(1.14%)	2(1.14%)
Immune-mediated pneumonia	0	0	1(0.57%)	1(0.57%)
Rash	7(9.21%)	1(1.31%)	8(4.57%)	1(0.57%)
Proteinuria	4(5.26%)	0	7(4.00)	0

*The incidence of any adverse event was not significantly different between the two groups (*P*=0.51).

#The incidence of 3–4 adverse event was not significantly different between the two groups (*P*=0.09).

The most common grade 3 or 4 adverse events in the HTP and HT groups were neutropenia (17/175, 9.71% vs 1/76, 1.32%), elevated aspartate transaminase or alanine aminotransferase (17/175, 9.71% vs 6/76, 7.89%), thrombocytopenia (5/175, 2.86% vs 1/76,1.32%). The most common adverse events observed in the HTP or HT group included elevated levels of aspartate transaminase or alanine aminotransferase (78/175, 44.57% vs 32/76, 42.11%), neutropenia (45/175, 25.71% vs 9/76, 11.84%), thrombocytopenia (52/175, 29.71% vs 22/76, 28.95%), hypoalbuminemia (37/175, 21.14% vs 16/76, 21.05%) and elevated bilirubin (20/175, 11.43% vs 6/76, 7.89%).

Two treatment-related deaths occurred in the HTP group, one due to immune hepatitis and the other due to immune pneumonia. The HT group without immunization showed no fatal complications.

## 4 Discussion

This retrospective study demonstrated that PD-1 inhibitor-augmented HAIC-TKI therapy in HCC with PVTT. The triple therapy achieved a superior median OS of 24.6 months versus 13.5 months in the dual therapy group. However, for HCC patients aged > 60 years or those with extra-hepatic metastases, the addition of PD-1 inhibitors did not yield statistically significant survival benefits. Notably, the safety profile remained comparable between groups, with no significant increase in treatment-related adverse events after PD-1 inhibitor incorporation.

The therapeutic efficacy of HAIC-FOLFOX combined with TKI alone for HCC with PVTT was suboptimal (15). The integration of PD-1 inhibitors into the HAIC-TKI regimen substantially enhanced clinical outcomes, extending median survival by 11.1 months compared to dual therapy. To date, no phase III clinical trials have validated this triple combination for HCC with PVTT (27, 28). However, there are ongoing related clinical trials, for example. Shi et al. designed a randomized controlled and double-blind trial to compared FOLFOX-HAIC plus lenvatinib and toripalimab vs. FOLFOX-HAIC plus lenvatinib for advanced HCC (ClinicalTrials.gov ID NCT06201065). Wang et al. (29) reported similar findings in a comparative study (n=76), where trans-arterial interventional therapy combined with TKIs and PD-1 inhibitors (n=39) demonstrated significantly longer median OS than dual therapy (16.1 vs 10.3 months, *P*=0.007). A meta-analysis by Du et al. (17) encompassing six cohort studies further corroborated these results, showing that triple therapy (interventional therapy + TKI + immune checkpoint inhibitors) significantly improved both OS (HR=0.63, *P*<0.00001; mean difference (MD)=5.08 months, *P*<0.001) and PFS (HR=0.46, *P*<0.0001; MD=3.42 months, *P*<0.001) versus dual therapy.

Significant advancements have been attained in the management of HCC with PVTT, resulting in a median survival exceeding 20 months (27, 28, 30). Nevertheless, HCC patients with PVTT Vp4 still had unsatisfying survival outcomes (10). In our cohort of 97 HCC patients with PVTT Vp4, the HTP group (HAIC-TKI-PD-1, n=29) showed a median OS of 15.3 months versus 9.3 months in the HT group (HAIC-TKI, n=26) (*P*=0.04). In a separate study, HCC patients with PVTT Vp4 who received HAIC-TKI-PD-1 inhibitor or HAIC-TKI had a median OS of 15.9 compared with 9.6 months (*P*=0.05), which was consistent with our findings (29). Notably, the IMbrave150 trial reported a non-significant OS difference (7.6 vs 5.5 months, *P*=0.104) between atezolizumab-bevacizumab and sorafenib in PVTT Vp4 patients (10). Given the high risk of gastrointestinal bleeding in PVTT Vp4 patients, HAIC-based regimens may offer safer therapeutic alternatives compared to systemic anti-angiogenic therapies for these patients.

Subgroup analysis revealed a marginal 1.9-month OS extension in patients >60 years (*P*=0.363), versus a 12.5-month improvement in younger patients (*P*=0.003). The immunosenescence phenomenon may explain diminished PD-1 inhibitor efficacy in elderly patients. Age-related declines in T-cell function, and particularly CD8+ cytotoxic activity likely contribute to this disparity (31, 32). Older patients also had a higher incidence of immune-related adverse events, which may affect their treatment tolerance (4). These findings underscore the need for personalized immunotherapy strategies in geriatric populations, incorporating comprehensive assessments of immune status and comorbidities (33–35). PD-1 inhibitors demonstrated limited efficacy in HCC patients with extra-hepatic metastases, extending OS by only 3.0 months (*P*=0.913) versus 17.3 months in non-metastatic cases (*P*=0.005). Guo et al (36) compared lenvatinib and PD-1 inhibitor with or without HAIC-FOLFOX for HCC patients with extrahepatic metastases, the HAIC-LEN-P group significantly extended the mOS and mPFS compared with LEN-P alone (mOS: 27.0 months vs. 9.0 months, *P* < 0.001; mPFS: 8.0 months vs 3.0 months, *P*=0.001). The ORR in the combination group was twice as high as that of the LEN-P group (67.3% vs 29.1%, *P* < 0.001). PD-1 inhibitors and TKI only achieved a median OS of 9.0 months without HAIC, which was constant with our results that PD-1 inhibitor was less effective in HCC patients with extrahepatic metastasis (37, 38).

Our study had several limitations. First, while the inclusion of diverse TKIs and PD-1 inhibitors reflects real-world clinical practice, this pharmacological heterogeneity may introduce confounding factors that could affect outcome comparability between therapeutic regimens. The varying efficacy profiles (e.g. sorafenib vs. lenvatinib) and safety characteristics among different agents might obscure the precise evaluation of individual drug contributions. Future studies should consider standardized therapeutic protocols with subgroup analyses stratified by specific agent types to better elucidate differential treatment effects. Second, the absence of PD-1 inhibitor administration following tumor progression in patients receiving HAIC combined with TKI therapy may potentially underestimate the full therapeutic potential of PD-1 inhibitors in HCC patients with PVTT. This design limitation restricts our understanding of sequential treatment strategies and combination therapy optimization. Prospective trials should incorporate protocol-defined salvage therapies with PD-1 inhibitors to systematically evaluate their role in different treatment sequences. Third, the limited statistical power in certain subgroup analyses, particularly those that did not reach significance, restricts our ability to detect clinically meaningful differences in specific patient populations. This insufficiency may mask important treatment-effect modifiers and hinder personalized therapeutic approaches. Lastly, this is a single-center retrospective study with inevitable selection bias. These limitations highlight the critical need for validation through multicenter studies or prospective trials.

## 5 Conclusion

The triple therapy (HAIC-TKI-PD-1) demonstrated superior efficacy over HAIC-TKI dual therapy in HCC patients with PVTT, achieving significant improvements in ORR (66.3% vs. 51.3%, *P*=0.034), mOS (24.6 vs 13.5 months, *P*=0.001), and mPFS (11.1 vs 6.4 months, *P*<0.001). PD-1 inhibitors showed minimal survival benefits in HCC patients aged >60 or with extrahepatic metastases. Multicenter studies or prospective trials are needed to validate the above viewpoints.

## Data Availability

The datasets presented in this article are not readily available because all data belongs to the hospital and requires the hospital’s consent. Requests to access the datasets should be directed to caofei@sysucc.org.cn.
