# Supplementary material for: PD-1 inhibitor-augmented HAIC-TKI therapy in hepatocellular carcinoma with portal vein tumor thrombosis: real-world survival benefits, safety, and subgroup-specific efficacy
Source: Front Immunol. 2025 Jun 12;16:1602031. doi: 10.3389/fimmu.2025.1602031 (PMC12198130; doi:10.3389/fimmu.2025.1602031)
Supplement: Supplementary file 1 [file DataSheet1.pdf]

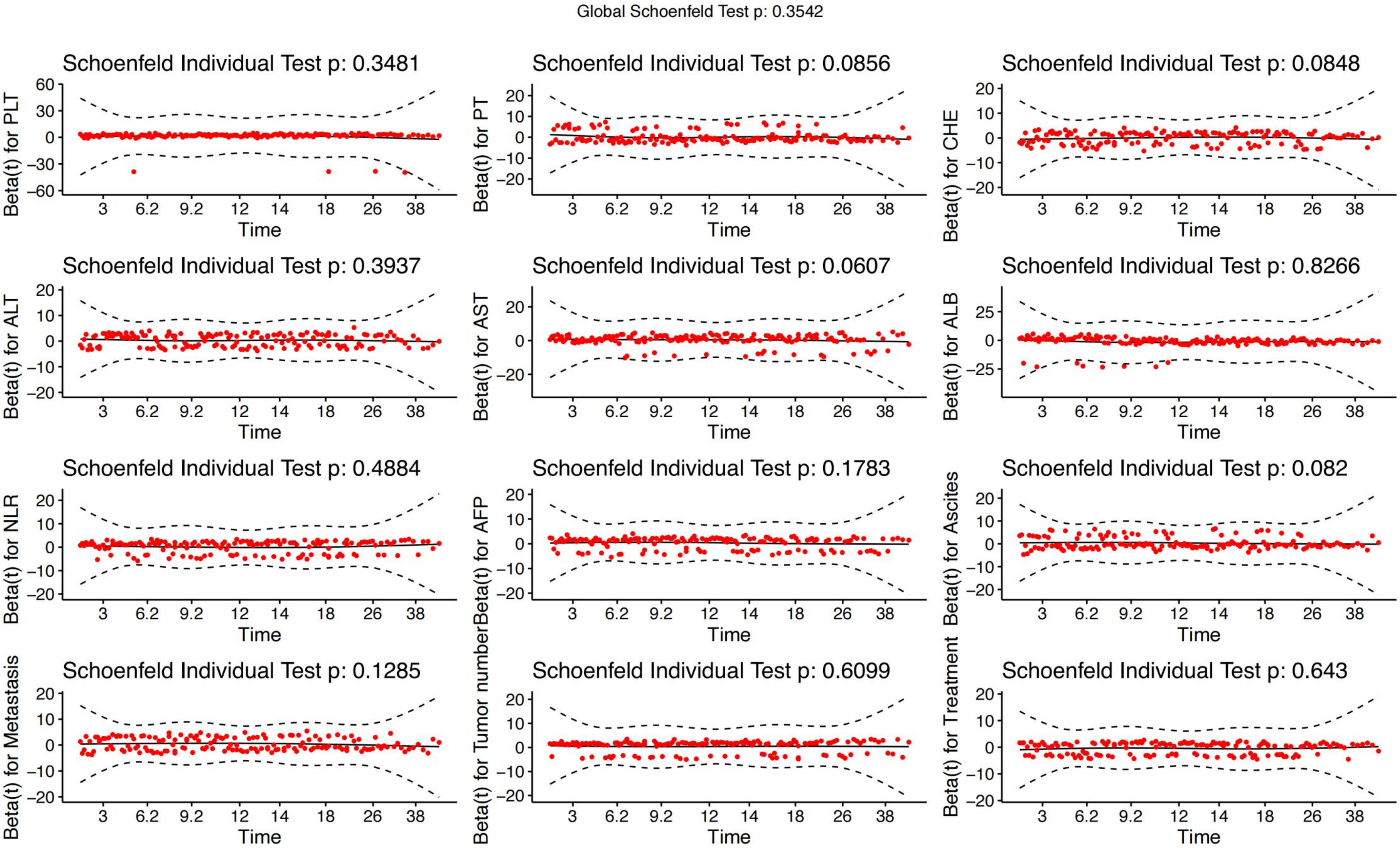

Supplementary Figure 1. Proportional hazards (PH) assumption validation for OS multivariate Cox regression models. Schoenfeld residual tests demonstrated no significant violations of the PH assumption

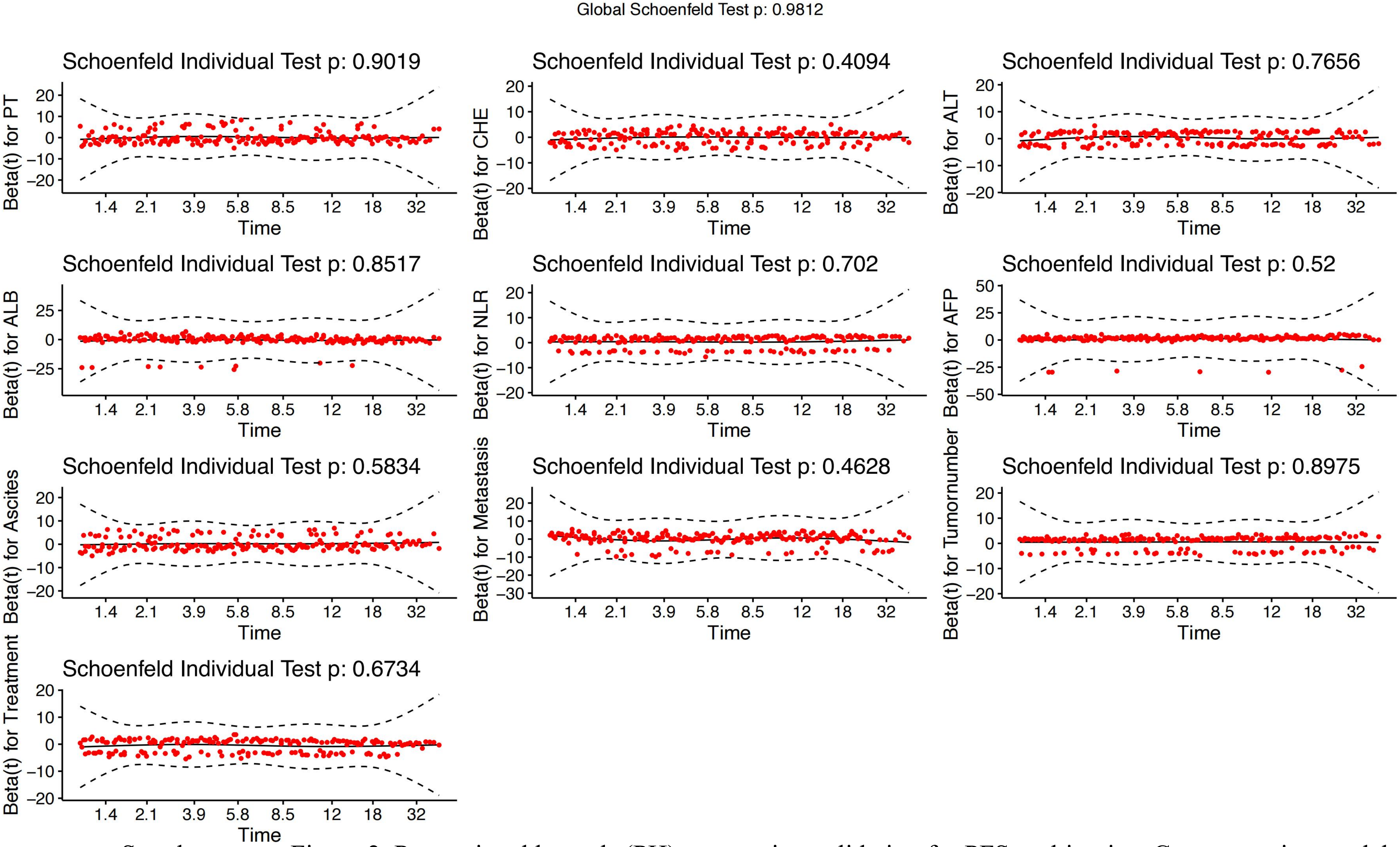

Supplementary Figure 2. Proportional hazards (PH) assumption validation for PFS multivariate Cox regression models. Schoenfeld residual tests demonstrated no significant violations of the PH assumption
